# Genetic polymorphism in *DGCR8* is associated with late onset of preeclampsia

**DOI:** 10.1186/s12881-019-0887-7

**Published:** 2019-09-04

**Authors:** Xin Huang, Zuodong Li, Jun Lei, Dapeng Wang, Yujing Zhang

**Affiliations:** 1 Department of Preventive Medicine, School of Medicine, Hunan Normal University, 371 Tongzipo Road, Yuelu District, Changsha, 410013 Hunan China; 2 Department of Obstetrics and Gynecology, The Third Xiangya Hospital of Central South University, Changsha, 410013 Hunan China; 3 Key Laboratory of Environmental Pollution Monitoring and Disease Control, Guizhou Medical University, Guiyang, 550025 Guizhou China

**Keywords:** MicroRNA machinery genes, Genetic susceptibility, *DGCR8*, Preeclampsia, Case control study

## Abstract

**Background:**

PE (preeclampsia) is a heterogeneous disorder with early onset PE (EOPE) and late onset PE (LOPE) subtypes. Associations between maternal miRNAs biosynthesis genes polymorphisms and risk of PE have been previously observed. However, the impact of polymorphisms in *DGCR8* which is indispensable in miRNA maturing processing on the susceptibility to preeclampsia (PE) has not been elucidated yet. We, therefore, conducted a case-control study to evaluate the impact of polymorphisms in *DGCR8* on the risk of EOPE and LOPE.

**Methods:**

A total of 66 patients diagnosed with EOPE, 206 with LOPE and 330 healthy controls were recruited. Five SNPs in *DGCR8* were genotyped including rs1558496, rs1640299, rs720012, rs720014, and rs9606241. Logistic regression was used to estimate the OR and the 95% CI for the associations.

**Results:**

Increased risk of LOPE has been observed among patients with rs1640299 TG genotype (OR = 1.98 (95%CI: 1.38, 2.87), *p* = 2.32e-4) and rs720014 TC genotype (OR = 2.49 (95%CI: 1.72, 3.60), *p* = 1.40e-7). The *DGCR8* rs1558496/ rs1640299/ rs720012/ rs720014/ rs9606241 haplotype T-G-A-C-A and T-G-A-C-G were associated with increased risk of LOPE (OR = 2.20 (95%CI: 1.49, 3.25), *p* = 5.90e-5, and 1.58 (95%CI: 1.06, 2.36), *p* = 0.024, respectively). And the haplotype T-T-G-T-A was associated with lower risk of LOPE (OR = 0.74 (95%CI: 0.58, 0.95), *p* = 0.018). These significant associations retained after false-positive discovery rate correction. However, none of the tested SNPs or haplotypes in *DGCR8* gene is associated with risk of EOPE (*p* > 0.05).

**Conclusions:**

Polymorphisms in *DGCR8* might participate in the pathological process of preeclampsia. The rs1640299 T > G and rs720014 T > C polymorphisms are associated with late onset preeclampsia susceptibility.

**Electronic supplementary material:**

The online version of this article (10.1186/s12881-019-0887-7) contains supplementary material, which is available to authorized users.

## Introduction

PE (preeclampsia) complicates 2–5% of pregnancies and is a major cause of maternal, fetal, and neonatal morbidity and mortality [1]. Furthermore, PE could increase the risk of long-term cardiovascular diseases of both mothers and their offspring [2, 3]. Though efforts have been made, the exact etiology for PE is still unclear.

MicroRNAs (miRNAs) have been proposed to regulate the stability of mRNAs that encode proteins involved in maintaining various normal physiological processes [4]. The biosynthesis of miRNAs is a tightly regulated multistep process. Single nucleotide polymorphisms (SNPs) within those miRNAs biosynthesis genes can result in altered miRNAs expression and subsequently being associated with risks of some diseases [5–7]. A recent study conducted in Iran has also observed the association between SNPs within maternal miRNAs biosynthesis genes and risk of PE [8].

DiGeorge critical region 8 (DGCR8) is an RNA binding protein which interacts with Drosha to produce pre-microRNA in the nucleus and is indispensable in miRNA maturing processing [9]. *DGCR8* also referred to as Pasha, could stabilize Drosha via protein- protein interaction, and take charge of recognizing ssRNA and dsRNA structures [10]. SNPs within the *DGCR8* are associated with schizophrenia [11], primary open angle glaucoma [12] and various cancers [13–15]. However, the impact of polymorphisms in *DGCR8 on the* susceptibility to PE has not been elucidated.

Moreover, PE is a heterogeneous disorder with 2 distinct subtypes: in most PE cases, the clinical features first come into view at or after 34 weeks of gestation called late onset PE (LOPE), and about 10% of cases showed the symptoms before 34 weeks of gestation called early onset PE (EOPE) [16]. Heterogeneous phenotypes in PE were resulted from different pathophysiological mechanisms [17, 18]. Decreased spiral artery remodeling and poor placentation are involved in EOPE. On the other hand, LOPE seems to be the manifestation of a mismatch between the metabolic demands of the growing fetus and maternal supply [17, 18]. Recent studies also found that different genetic predisposition also existed between LOPE and EOPE [19, 20]. We, therefore, have genotyped 5 SNPs in *DGCR8* including rs1558496, rs1640299, rs720012, rs720014, and rs9606241 in a case-control study to evaluate the impact of these genes polymorphisms on the risk of EOPE and LOPE.

## Methods

### Study design

A case-control study that included 66 EOPE, 206 LOPE and 330 controls was conducted between Mar 2016 and Dec2017 at two hospitals in Hunan province of China (the Liuyang Municipal Hospital of Maternal and Child Health (LYMHMCH), and the Third Xiangya Hospital of Central South University (TXYHCSU)). The inclusion criteria for participants have been depicted in our previous study [21]. In brief, cases were clinical diagnosed PE patients combined with absence of diabetes mellitus, renal disease, or chronic cardiovascular diseases. All the controls were confirmed with normal blood pressure during pregnancy and with no history of aforementioned chronic diseases.

### Clinical definitions

The diagnostic criteria for PE was de novo hypertension (≥140/90 mmHg) after 20 weeks of gestation accompanied with proteinuria (≥ 1+ on dipstick), or without proteinuria but showed one or more of the following symptoms: thrombocytopenia, impaired liver function, de novo renal insufficiency, pulmonary edema, cerebral or visual disturbance. PE was further classified as EOPE (diagnosed before 34 weeks of gestation), or LOPE (diagnosed at or after 34 weeks of gestation).

### Information collection and genotyping

The study protocol was approved by Ethical Review Committee of the Central South University and IRB (institutional review boards) from LYMHMCH. Written informed consent was obtained from all participants. A self-administered questionnaire was used to collect demographic, reproductive and medical history within two days before or after delivery. Meanwhile, 5 ml of venous blood sample from each participant was collected and processed for genomic DNA extraction with the TIANamp blood DNA kit (DP318–03, TIANGEN, Beijing) within 24 h. Information on maternal complications was abstracted from the medical records. Five candidate SNPs in *DGCR8* were selected and genotyped with the SEQUENOM MassARRAY iPLEX platform, including rs1558496, rs1640299, rs720012, rs720014, and rs9606241. Information on location and potential functions of selected SNPs were listed in Additional file 1: Table S1.

### Statistical analysis

χ^2^ test was conducted for comparison on maternal characteristics between EOPE, LOPE and control groups. The χ^2^ partition method (α^’^ = α/ (3*(3–1)/2) + 1) =0.0125) was used for further comparison between each of the two groups. Hardy-Weinberg equilibrium was detected in control group. The risk of LOPE and EOPE associated with genotypes or haplotype frequencies was calculated as the odds ratio (OR) with 95% confidence interval (95%CI) by unconditional logistic regression model. The false-positive discovery rate (FDR) correction was adopted to adjust multiple comparison tests. Two-tailed *p* values < 0.05 were considered statistically significant. Statistical analyses were performed with SAS software, version 9.2 (SAS Institute, Inc., Cary, NC). The impact of these selected SNPs on the expression of the proteins was further evaluated through Ensembl’s Variant Effect Predictor (VEP) tool (http://grch37.ensembl.org/info/docs/tools/vep/index.html).

## Results

General information on enrolled cases and controls were all listed in Table 1. Both EOPE and LOPE cases were older than controls. Compared to LOPE cases and controls, the EOPE cases were more likely to be nulliparous and exposure to active and/or passive smoking during pregnancy. And LOPE cases had higher risk of GDM than EOPE and controls. No significant differences in the distribution of maternal education, or fetal gender were found between EOPE, LOPE and controls.

**Table 1:** Distributions of characteristics between selected PE patients and Controls

Characteristics	Control (*n* = 330, (%))	EOPE (*n* = 66, (%))	LOPE (*n* = 206, (%))	*p*
Maternal age, years				
< 25	44(13.33) ^a^	6(9.09) ^a^	26(12.62) ^a^	< 0.001
25–30	177(53.64)	24(36.36)	66(32.04)	
> 30	109(33.03)	36(54.55)	114(55.34)	
Education, years				
≤ 12	118(35.76)	26(39.39)	90(43.69)	0.186
> 12	212	40(60.61)	116(56.31)	
Nulliparous				
Yes	152 (46.06) ^b^	42(63.64) ^b^	96(46.60) ^b^	0.028
No	178 (53.94)	24(36.36)	106(53.40)	
Active and/or passive smoking				
Yes	102 (30.90) ^b^	31(46.97) ^b^	61(29.61) ^b^	0.024
No	228 (69.09)	35(53.03)	145(70.39)	
Fetal gender				
Male	167 (50.61)	36(54.55)	86(41.75)	0.072
Female	163 (49.39)	30(45.45)	120(58.25)	
GDM				
Yes	17 (5.15) ^c^	6(9.09) ^c^	40(19.42) ^c^	< 0.001
No	313 (94.85)	60(90.91)	166(80.58)	

^a^Control compared with EOPE, *p* < 0.0125, control compared with LOPE, *p* < 0.0125, EOPE compared with LOPE, *p* > 0.0125;

^b^Control compared with EOPE, *p* < 0.0125, control compared with LOPE, *p* > 0.0125, EOPE compared with LOPE, *p* < 0.0125;

^c^Control compared with EOPE, *p* > 0.0125, control compared with LOPE, *p* < 0.0125, EOPE compared with LOPE, *p* < 0.0125;

Abbreviation: *PE* preeclampsia, *EOPE* early onset preeclampsia, *LOPE* late onset preeclampsia, *GDM* Gestational Diabetes Mellitus

The genotyping completion rates were 100% for all SNPs. All of the selected genes have showed polymorphisms and observed with HWE in the control group. Results from VEP showed that all of the 5 selected SNPs had a predicted ‘modifier’ impact (Additional file 1: Table S1).Table 2 showed the odds ratios for 5 selected SNPs and PE subtypes. After the FDR correction, increased risk of LOPE has been still observed among patients with rs1640299 TG genotype (OR = 1.98 (95%CI: 1.38, 2.87), *p* = 2.32e-4) and rs720014 TC genotype (OR = 2.49 (95%CI: 1.72, 3.60), *p* = 1.40e-7). However, none of the tested SNPs was associated with risk of EOPE (*p* > 0.05). Compared with controls, though the frequencies of rs720012 AA genotype were higher in LOPE group, this association became insignificant after FDR correction (*p* = 0.067).

**Table 2:** Associations between the polymorphisms in *DGCR8* gene and preeclampsia subtypes

Genotype	Controls	EOPE				LOPE			
	N (%)	N (%)	OR (95%CI)	*p*	*p* ^a^	N (%)	OR (95%CI)	*p*	*p* ^a^
rs1558496									
TT	288(87.27)	54(81.82)	1.00			185(89.81)	1.00		
TC	40(12.12)	10(15.15)	1.33(0.63, 2.83)	0.453	0.755	20(9.71)	0.78(0.44, 1.37)	0.387	0.484
CC	2(0.61)	2(3.03)	5.33(0.74, 38.69)	0.097	0.485	1(0.49)	0.78(0.07, 8.64)	0.838	0.838
rs1640299									
TT	195(59.09)	38(57.58)	1.00			85(41.26)	1.00		
TG	120(36.36)	23(34.85)	0.98(0.56, 1.73)	0.954	0.979	104(50.49)	1.98(1.38, 2.87)	2.32e-4	5.80e-4
GG	15(4.55)	5(7.58)	1.71(0.59, 4.99)	0.325	0.530	17(8.25)	2.60(1.24, 5.45)	0.011	0.055
rs720012									
GG	64(19.39)	14(21.21)	1.00			45(21.84)	1.00		
GA	154(46.67)	34(51.52)	1.00(0.51, 2.00)	0.979	0.979	115(55.83)	1.06(0.68, 1.67)	0.794	0.794
AA	112(33.94)	18(27.27)	0.74(0.34, 1.58)	0.428	0.530	46(22.33)	0.58(0.35, 0.98)	0.040	0.067
rs720014									
TT	231(70.00)	50(75.76)	1.00			99(48.06)	1.00		
TC	91(27.58)	13(19.70)	0.66(0.34, 1.27)	0.215	0.538	97(47.09)	2.49(1.72, 3.60)	1.40e-7	7.00e-07
CC	8(2.42)	3(4.55)	1.73(0.44, 6.76)	0.429	0.530	10(4.85)	2.92(1.12, 7.61)	0.0287	0.067
rs9606241									
AA	240(72.73)	41(62.12)	1.00			139(67.48)	1.00		
AG	83(25.15)	23(34.85)	1.62(0.92, 2.86)	0.095	0.475	60(29.13)	1.25(0.84, 1.85)	0.268	0.447
GG	7(2.12)	2(3.03)	1.67(0.34, 8.33)	0.530	0.530	7(3.40)	1.27(0.59, 5.03)	0.316	0.395

^a^ FDR adjusted *p* value;

Abbreviation: *EOPE* early onset preeclampsia, *LOPE* late onset preeclampsia

The associations between possible haplotypes and risk of EOPE and LOPE were estimated and listed in Table 3. The *DGCR8* rs1558496/ rs1640299/ rs720012/ rs720014/ rs9606241 haplotype T-G-A-C-A and T-G-A-C-G were significantly associated with increased risk of LOPE (OR = 2.20 (95%CI: 1.49, 3.25) and 1.58 (95%CI: 1.06, 2.36), respectively). However, the haplotype T-T-G-T-A was associated with lower risk of LOPE (OR = 0.74 (95%CI: 0.58, 0.95)). Those associations retained after FDR correction (*p* < 0.05).

**Table 3:** Associations between haplotypes of *DGCR8* genes and preeclampsia subtypes ^a^

Haplotype	Control	EOPE			LOPE				
	freq	freq	OR(95%CI)	*p*	*p* ^*b*^	freq	OR(95%CI)	*p*	*p* ^*b*^
*DGCR8* rs1558496/ rs1640299/ rs720012/ rs720014/ rs9606241									
C G A T G	0.063	0.106	1.74(0.92, 3.30)	0.083	0.415	0.053	0.83 (0.49, 1.42)	0.495	0.495
T G A C A	0.077	0.045	0.57(0.24, 1.35)	0.195	0.488	0.155	2.20 (1.49, 3.25)	5.90e-5	2.95e-4
T G A C G	0.084	0.098	1.19 (0.63, 2.25)	0.588	0.597,	0.126	1.58 (1.06, 2.36)	0.024	0.040
T T A T A	0.198	0.220	1.13 (0.72, 1.78)	0.597	0.597	0.163	0.78 (0.57, 1.08)	0.136	0.170
T T G T A	0.573	0.530	0.83(0.57, 1.21)	0.339	0.565	0.500	0.74 (0.58, 0.95)	0.018	0.040

^a^All those frequency < 0.03 were ignored in analysis;

^b^FDR adjusted *p* value;

Abbreviation: *EOPE* early onset preeclampsia, *LOPE* late onset preeclampsia

## Discussion

In our study, we have evaluated five SNPs (rs1558496, rs1640299, rs720012, rs720014 and rs9606241) of the *DGCR8* gene with the risk of EOPE and LOPE in Han-Chinese population. The results showed that rs1640299 genotype TG and rs720014 genotype TC were associated with increased risk of LOPE. *DGCR8* haplotypes are also significantly associated with risk of LOPE. However, none of the tested SNPs or haplotypes in *DGCR8* gene is associated with risk of EOPE.

Generation of pre-miRNAs from the longer pri-miRNAs by the microprocessor is the first step of miRNAs maturation [9]. As a key component of microprocessor, DGCR8 takes charge of stabilizing Drosha via protein- protein interaction, recognizing the pri-miRNA at the ssRNA-dsRNA junction and directing Drosha to a specific cleavage site [10]. It is indispensable for miRNAs biogenesis. Emerging evidence has supported that the genetic variations within *DGCR8* were associated with various diseases [11–15]. The polymorphism in rs1640299 was reported to be associated with risk of laryngeal cancer [13] and bladder cancer [14], and could increase the effect of black carbon pollutant exposures on blood pressure (BP) [22]. In our study, we also found that compared with wild genotype, the risk of LOPE among rs1640299 TG genotype carriers was increased by almost 2 fold (OR = 1.98 (95%CI: 1.38, 2.87)). Moreover, patients with rs720014 TC genotype had a 2.49 fold increased risk of LOPE (OR = 2.49 (95%CI: 1.72, 3.60)). Previous studies conducted in Caucasians also found that ovarian cancer survival [23] and radiotherapy induced pneumonitis in patients with non-small cell lung cancer [24] were significantly associated with polymorphism in rs720014, but not associated with rs720012 polymorphism which is in consist with our findings. The polymorphisms in rs1640299 and rs720014 were both located at the 3′UTR of *DGCR8* which is not translated directly into the protein. However, based on the bioinformatic prediction (http://snpinfo.niehs.nih.gov/index.html), rs1640299 is located at the binding sites of miR-1256 and miR-583 in the 3′UTR of *DGCR,* and rs720014 is located at the binding sites of miR-106, miR-17, miR-20, miR-518, miR-770 and miR-93. Mutations on those two 3′UTR sites might lead to impairment of those miRNAs binding and thereby modify the process of miRNAs maturation and consequently may play a pivotal role in PE. Additionally, impairment of miRNAs binding could also affect mRNA stability and lead to upgraded DGCR8 expression which has been previously observed in Iranian PE patients [25]. However, the specific molecular mechanisms of those two SNPs need further experimental validation.

The impact of polymorphisms in rs9606248 and rs1558496 on susceptibility to diseases has been previously tested in breast cancer [26] and colon cancer [27]. And no significant association has been observed. Our results also suggest no significant association existing between those two SNPs and EOPE/ LOPE.

Growing evidence proved that LOPE and EOPE are two distinct phenotypes of PE, which were resulted from different pathophysiological mechanisms [17, 18]. Decreased spiral artery remodeling and poor placentation are involved in EOPE. On the other hand, LOPE seems to be the manifestation of a mismatch between the metabolic demands of the growing fetus and maternal supply [17, 18]. Recently, studies conducted in Australia and Brazilian found that maternal polymorphisms in *HIF1α* [19] and *ACVR2A* [20] genes were associated with risk of EOPE, but not with LOPE. Those findings implied that different genetic predisposition existed between LOPE and EOPE. Our study added evidence to this hypothesis: *DGCR8* polymorphisms are only associated with LOPE susceptibility. The *DGCR8* gene plays a central role in angiogenesis and cardiovascular function [28, 29]: deletion of *DGCR8* in cardiomyocytes could lead to the increase of angiogenesis genes and left ventricular malfunction. The angiogenic/anti-angiogenic imbalance is milder in LOPE rather than in EOPE [30]. We speculated that the polymorphisms in rs1640299 and rs720014 may only cause mild angiogenic/anti-angiogenic imbalance which result different genetic predisposition observed in our study.

Strengths and limitations should be considered when interpreting the study findings. Diagnosis of EOPE/ LOPE was based on medical records not self-report, which minimized potential disease misclassification. However, the limitation should not be ignored as well. Our case control study only explores the association between SNPs in *DGCR8* and EOPE/ LOPE development, not the causal relationship. And the functional study of those SNPs to the development of LOPE has not been conducted in present study. Moreover, our study was conducted among Chinese with relative small sample size. The replication of our finding in an independent population is needed.

## Conclusion

Our studies confirmed that polymorphisms in *DGCR8* might participate in the pathological process of preeclampsia. The rs1640299 T > G and rs720014 T > C polymorphisms are associated with late onset preeclampsia susceptibility. However, the polymorphisms in *DGCR8* are not associated with risk of early onset preeclampsia. The mechanism on how those SNP modifies the susceptibility to pregnancy induced hypertension needs further research.

## Additional file

Additional file 1: **Table S1.** SNPs evaluated in this study and their minor allele frequencies (MAF) in the Han-Chinese population. (DOCX 17 kb)

## Data Availability

The datasets used and analyzed during the current study are available from the corresponding author on reasonable request.

## Abbreviations

DGCR8: DiGeorge critical region 8
EOPE: Early onset preeclampsia
FDR: False-positive discovery rate
GDM: Gestational diabetes mellitus
LOPE: Late onset preeclampsia
miRNA: microRNA
PE: Preeclampsia
SNPs: Single nucleotide polymorphisms
VEP: Variant Effect Predictor
