# Supplementary material for: Genetic polymorphism in DGCR8 is associated with late onset of preeclampsia
Source: BMC Med Genet. 2019 Sep 4;20:151. doi: 10.1186/s12881-019-0887-7 (PMC6727569; doi:10.1186/s12881-019-0887-7)
Supplement: Supplementary file 1 — Table S1. SNPs evaluated in this study and their minor allele frequencies (MAF) in the Han-Chinese population. (DOCX 17 kb) [file 12881_2019_887_MOESM1_ESM.docx]

Table S1. SNPs evaluated in this study and their minor allele frequencies (MAF) in the Han-Chinese population

| Polymorphisms | Functional Consequence | Effect allele | Other allele | MAF (%)* | VEP Prediction |
| --- | --- | --- | --- | --- | --- |
| rs1558496 | upstream variant | T | C | 0.134 | modifier |
| rs1640299 | utr variant 3 prime | T | G | 0.233 | modifier |
| rs720012 | utr variant 3 prime | G | A | 0.426 | modifier |
| rs720014 | utr variant 3 prime | T | C | 0.219 | modifier |
| rs9606241 | upstream variant | A | G | 0.231 | modifier |

*MAF in Han-Chinese population, as reported in dbSNP database.
